# Investigating Semantic Augmentation in Virtual Environments for Image Segmentation Using Convolutional Neural Networks

**DOI:** 10.3390/jimaging7080146

**Published:** 2021-08-14

**Authors:** Joshua Ganter, Simon Löffler, Ron Metzger, Katharina Ußling, Christoph Müller

**Affiliations:** 1 Faculty of Digital Media, Furtwangen University, 78120 Furtwangen, Germany; joshua.michael.ganter@hs-furtwangen.de (J.G.); s.loeffler@hs-furtwangen.de (S.L.); ron.marvin.metzger@hs-furtwangen.de (R.M.); katharina.ussling@hs-furtwangen.de (K.U.); 2 Fraunhofer-Institute for Physical Measurement Techniques IPM, 79110 Freiburg, Germany

**Keywords:** semantic augmentation, image segmentation, convolutional neural networks, virtual image data

## Abstract

Collecting real-world data for the training of neural networks is enormously time-consuming and expensive. As such, the concept of virtualizing the domain and creating synthetic data has been analyzed in many instances. This virtualization offers many possibilities of changing the domain, and with that, enabling the relatively fast creation of data. It also offers the chance to enhance necessary augmentations with additional semantic information when compared with conventional augmentation methods. This raises the question of whether such semantic changes, which can be seen as augmentations of the virtual domain, contribute to better results for neural networks, when trained with data augmented this way. In this paper, a virtual dataset is presented, including semantic augmentations and automatically generated annotations, as well as a comparison between semantic and conventional augmentation for image data. It is determined that the results differ only marginally for neural network models trained with the two augmentation approaches.

## 1. Introduction

With the rise of convolutional neural networks, computer vision tasks became solvable with deep learning approaches. In particular, image classification became precise and reliable at a reasonable computational expense. Semantic segmentation, a flavor of image classification that performs a per-pixel classification, is used heavily in various areas, including scene understanding for autonomous driving [1,2].

The goal of this per-pixel classification is to gain an understanding of the scene’s semantics. With multiple neighboring pixels representing the same class, areas can be defined where one or more objects of the respective class are located. This information can be analyzed further: If the point of view is known, a processor can calculate the 3D location of the object(s) and decide on a course of action. In this work, for example, the recognition of the environment based on images provided by a camera attached to the front of a train is investigated.

Different instances of objects representing the same class are not differentiated. Therefore, it is not possible to distinguish my dog from your dog if dog is a class. For solving such a task, instance segmentation is used. Still, semantic segmentation suffices for many use-cases. Semantic segmentation is mostly implemented with deep convolutional neural networks (DCNNs). For the training of a DCNN, a huge amount of training data is needed. Furthermore, the training data needs to contain diversity in order to mitigate the effect of overfitting. Since acquiring huge amounts of training data is often very expensive or even impossible, the available data are augmented.

The method described in this paper relates to supervised neural networks applied to image segmentation, where the generation of training data by humans is a time-consuming and costly process. Varying approaches exist to reduce the amount of manual work to generate training data, such as synthetically generated data or augmentation of existing data.

Augmentation is used massively throughout different fields of data sciences to artificially enlarge datasets. There are multiple approaches to augmentation for different fields of application. Many algorithms exist to manipulate data at the pixel level. A popular library containing a range of such algorithms is imgaug [3]. Furthermore, Generative Adversarial Networks (GANs) [4] are capable of generating additional variations based on the original data.

**Figure 1:**
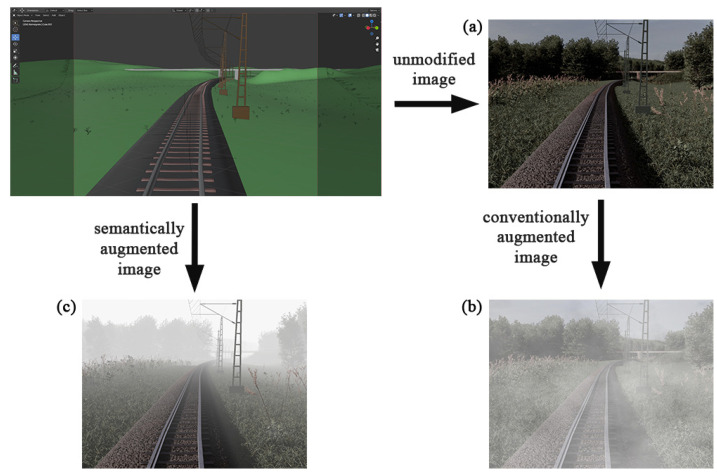
Comparison of original image (**a**), conventionally augmented image (**b**) and semantically augmented image (**c**); all created synthetically.

Instead of generating new data with GANs or modifying existing data on a signal-level only, it is possible to take the semantics of the data into account. Data for a neural network that is to be trained on detecting forest scenes could be augmented on a pixel-level simply by changing the hue for all pixels, or on a semantic level by changing the leaves’ color to brown. An example of semantic augmentation compared to conventional augmentation with fog is shown in Figure 1.

For the change of a semantic level, the following term is defined for this work: Semantic augmentation is a type of data augmentation, which is applied in a contextually correct manner on a semantic abstraction layer. Properties that fulfill the requirements of contextual and semantic correctness have to be defined separately for different domains, as not every imaginable semantic augmentation makes sense in a given context. As an implication of the above definition, the implemented augmentation technique needs to be aware of the context and semantics of the data. Consequently, algorithms for semantic augmentation on real image data would need to understand the semantics of the image content. This yields a conflict since the machine learning model is trained to solve that exact problem in the first place. Therefore, semantic augmentation of real image data is highly impractical.

This paper compares two approaches of generating augmentations on existing data: semantic and conventional augmentation. Semantic augmentation as defined above can only be applied to synthetically generated training data; thus, the comparison of the two augmentation approaches is entirely performed on training data based on computer-generated images. This paper does not cover a comparison between manually generated training data based on real images and synthetically generated training data based on computer-generated images, nor does it cover an assessment of the performance of synthetic training data on different neural network architectures. It also does not cover a comparison between fundamental neural network approaches, such as supervised versus unsupervised learning.

With the scene composition digitally available, the task of making semantically correct modifications becomes manageable, while still imposing challenges, which are discussed later in this paper.

### 1.1. Terminology

1. The (augmentation) **type** determines the technical approach of how the image data are augmented. The augmentation type may be
  - Conventional augmentation for approaches that are used broadly and are classified as Basic Image Manipulations by Shorten and Khoshgoftaar [5] or
  - Semantic augmentation for approaches described in the previous section.
2. The (augmentation) **category** determines the semantic operation that is applied to the image data for augmentation, with examples being snowflakes or rain. The category does not define the actual implementation used to achieve the semantic operation, as these differ between the augmentation types.
3. The (augmentation) **strength** determines the magnitude of change the augmentation methods have on the image data. Weak, weak-medium, medium, medium-strong and strong are used as adjectives in ascending order of magnitude. Each augmentation strength contains the parameters for each augmentation category in each augmentation type.

### 1.2. Structure

The structure of this paper is as follows: Section 2 first describes existing research related to the topic of semantic segmentation and augmentation. Next, Section 3 defines and describes the research methods that are used in this paper. Section 4 presents the results and explains problems that have arisen. Finally, Section 5 concludes and discusses future issues.

## 2. Related Works

Artificial neural networks have been used for semantic image segmentation in many application areas. In the paper by Liu et al. [1], in addition to a brief summary of traditional methods for semantic segmentation, recent advances in semantic image segmentation using Deep Convolutional Neural Networks (DCNNs) are described. Related works on the same topic include [6,7,8,9,10,11]. For example, the authors of the paper [6] aim to improve the accuracy of pixel classification in semantic segmentation by developing a new object context scheme that explicitly extends object information. Another topic that is frequently investigated is the methods that can be used to improve the structure of the neural network with respect to semantic segmentation. In the work of [7], an innovative structure is developed to replace the standard convolutional layers with square kernels. In [8], a specialized graph neural network (GNN) is developed to capture contextual information using a criss-cross path, and in [9], the design of the semantic segmentation model is rethought and an alternative is offered. In particular, the authors of [9] propose to replace the encoder based on stacked convolutional layers with a pure transformer.

This work does not investigate the semantic segmentation process itself, nor the neural network architectures used for this purpose, but rather investigates a way to extend the dataset to train a network for image recognition using semantic segmentation.

The training of DCNNs for semantic segmentation requires a large number of training data. In classical approaches, real images are used for these training data as in [12,13]. The work of Zendel et al. [12] presents a dataset consisting of imagery taken from a train engine’s driver cab extracted from video sequences of various train runs. The annotation of the image regions is created here by a combination of manually created geometric shapes as well as weakly supervised annotations generated with semantic segmentation networks from the road domain. Furthermore, in [13], a dataset for train runs is created from video files. Here, the annotation is implemented using manual labeling and is specifically implemented for the signal signs in France.

Training data can also be generated from synthetically generated data. In 2016, Gaidon et al. [14] presented the artificial dataset “Virtual KITTI”. In this dataset for the road domain, consistent ground-truth annotations are automatically generated for each video sequence. A special advantage of this dataset is the possibility of changing the weather of the scenes (e.g., to generate fog or similar). The authors describe that the subject of data augmentation using such semantic modification could be further explored for building more robust models. This dataset was improved in 2020 by Cabon et al. [15] by setting up a stereo camera (dataset “Virtual KITTI 2”); however, data augmentation is not considered further here.

In order to expand the amount of training data and make it more diverse, the training images can be augmented using various methods. The work of Shorten and Khoshgoftaar [5] summarizes possible methods of augmentation for training data. Among the conventional augmentations, the basic image manipulations are mentioned, which correspond to the following image processing methods: kernel filters, color space transformation, random erasing, geometric transformations, mixing images. For deep learning approaches, the methods Adversarial Training, Neural Style Transfer and GAN Data Augmentation are explained. In addition, combinations of basic image manipulations and deep learning approaches are also mentioned in [5], but semantic augmentations are not discussed further.

The methodology of semantic augmentation is the focus of this work, since it is rarely used at present. As described earlier, this augmentation technique is mentioned in [14] but not discussed in detail. In the area of text recognition, semantic augmentation is successfully applied in the works [16,17]. In [18], a deep learning approach is used for semantic augmentation. Here, an algorithm is developed that performs semantic augmentation of the data for training deep image recognition networks. The algorithm shifts feature in different directions in the deep feature space to create new semantic feature representations. No image data are generated, but the feature space is accessed directly to perform the semantic augmentations. Wood [19] shows how synthetic data can be created with digital humans. The “digital wardrobe” mentioned here can be understood as a semantic augmentation because, for example, from a single representation of a human hand, many different looking hands can be created. By adjusting skin color, clothing, and adding accessories, a new series of training data is created from one single view.

In this work, the approaches described in [18] and shown in [19] are considered in more detail. The developed method can be seen as another way to increase the size of datasets, since by semantically modifying the scene (such as creating snow on the scene objects), a massive growth of diversity compared to the original scene can be achieved.

## 3. Methodology

This section describes how the research proceeded. The basic idea is to investigate the impact of semantic augmentation of synthetic image data on training an artificial neural network for semantic segmentation compared to conventional augmentation. For this study, a 3D environment is modeled, which is used to generate synthetic training data. In the following, it is described how this 3D environment is created and what advantages it offers.

**Figure 2:**
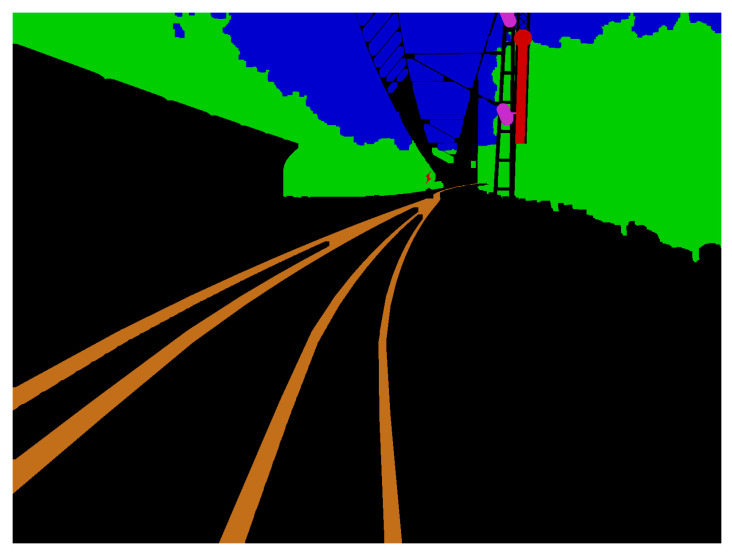
Example of an annotated image.

The created 3D environment is set up in the context of a train run in Germany. It is modeled and rendered using the tool blender. By creating the 3D world manually, the appropriate annotation images can also be generated automatically, parallel to rendering images of the scene. Such an annotation, corresponding to a rendered image, is shown in Figure 2. For the generation of the annotation images, the blender’s internal functions are used, namely object and material IDs. With this technique, an ID can be assigned to an object or material. In an additional render pass, these IDs can be written in a separate layer. The resulting maps act as object and material masks. The same ID is assigned to all objects or materials that are part of entities of the same class. This way, the object and material masks represent class masks. These masks are colorized, post-processed and layered on top of each other from background to foreground. The post-processing is performed to prevent too fine “pixelperfect” annotations and holes in labeled objects in the foreground.

Once the 3D environment is modeled, the next step is to determine how different training datasets can be created in order to compare conventional and semantic augmentation. For this purpose, different augmentation categories are defined. The categories need to match the following requirements:

1. They must be implementable with both augmentation types.
2. They should make sense in the specific domain, which means they can also appear in the real data.
3. They should be comparable between both augmentation types.

As mentioned before, in addition to the augmentation categories, different levels of augmentation strengths are determined. This is crucial to gain knowledge about how strong an augmentation must be in order to obtain good results in the test. Further details on the topic of augmentation categories and strengths will be explained later.

In a first iteration after the decision on the augmentation categories and strengths, the synthetic dataset of this work was augmented conventionally with imgaug [3] using parameters that provide realistic results. The parameters used are listed in Supplemental Material Table S1. Simultaneously, the implementations of semantic augmentations were completed in a blender. The semantically augmented image data should be generated in such a way that both augmentations represent a comparable difference from the original image. For this comparability, a similarity score, using the Learned Perceptual Image Patch Similarity (LPIPS), was calculated.

In the following, the LPIPS metric is explained and the creation of the datasets and augmentations is described. Afterward, the network architecture is explained, as well as how the training was implemented.

### 3.1. Similarity Score—LPIPS

LPIPS is described by Zhang et al. [20] and uses activations of a neural network to determine image similarity. In their findings, this method yields much better results than previous algorithms used to calculate perceived image similarity. These results apply to different network models. After feeding image data, a similarity score (LPIPS score) is calculated between two images. The lower the LPIPS is, the more similar the two compared images are. This is used by calculating the similarity score between the unaugmented picture (1) and the conventionally augmented picture (2). The generated semantically augmented picture (3) is then optimized to match the similarity score between pictures (1) and (2). If both similarity scores are roughly the same, it proves that both augmentations modified the image content by a comparable amount. The optimizations of the parameters for the semantic augmentations are to be determined in several iterations.

### 3.2. Datasets

All image data generated in the same augmentation type and belonging to an augmentation strength form a dataset. For example, a dataset is “conventional strong”. The original, unaugmented image data were added to each dataset. The selected network was trained in distinct training sessions with the datasets. Subsequently, these trained models were compared to each other on the test dataset using the Intersection over Union (IoU) metric and the LPIPS.

The dataset, formed by real image data, was provided by the Fraunhofer Institute for Physical Measurement Techniques (IPM). The dataset is situated in the domain of train tracks in Germany. It consists of 487 frames, each of which is coarsely annotated. There are six classes defined; these are:

1. Unknown,
2. Sky,
3. Vegetation,
4. Light signal,
5. Static signal,
6. Rail.

These classes were adopted for the synthetic datasets in this work.

**Figure 3:**
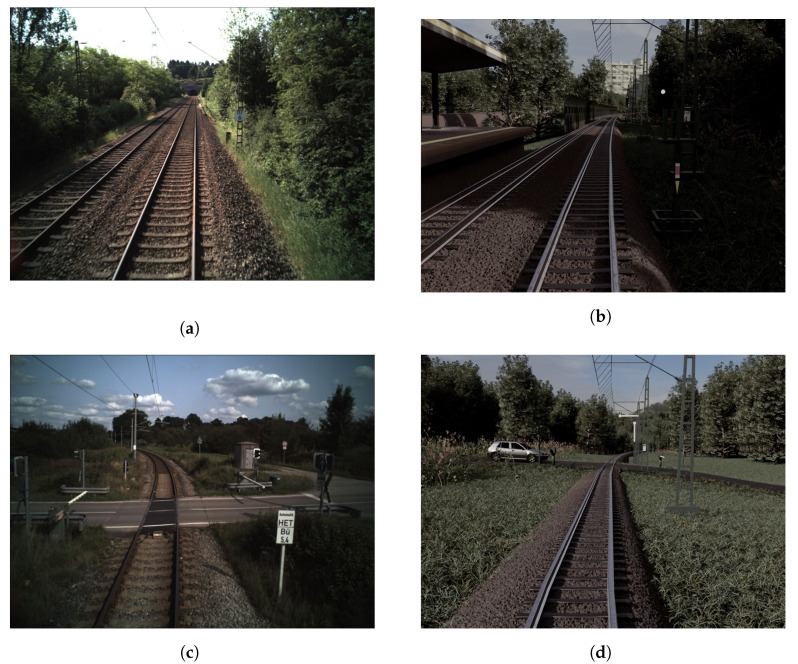
Real (**a**,**c**) and synthetic (**b**,**d**) data samples.

Despite the availability of several other datasets, it was decided to use the Fraunhofer dataset because it was captured on german railroad tracks. Two images of this real dataset can be seen on the left in Figure 3. Since railroad networks and signals hugely differ between countries, the trained network models probably cannot be used on data acquired in other countries. Additionally, the Fraunhofer dataset only contains a few classes compared to RailSem19 [12] or Virtual KITTI 2 [15], which simplifies the generation of synthetic data.

The model of this work was trained on synthetic data, and validation was performed on real data. Two synthetic images can be seen on the right in Figure 3. Real data were used for validation in order to mitigate possible side-effects imposed by generating our own benchmarks, where the data may be biased towards semantic or conventional augmentation. Testing was performed on real and synthetic data separately.

While creating the three-dimensional world, the real data were matched as closely as it was feasible. To show this, two synthetic and two real images are opposed in Figure 3. Several prominent visual characteristics of the original were recreated. The synthetic dataset features train stations, street crossings, other vehicles and trains, bridges, waters, trees and meadows—resembling the diversity in the underlying real data. Camera parameters such as height and focal length were adopted. The colors were manipulated to appear as in the original data. To enlarge the total amount of rendered images without increasing the size of the three dimensional world, images were taken with the train moving in both directions along the track.

The synthetic data forms a set with 251 frames in the forward direction and 251 frames in the backward direction, summing up to 502 frames, with fine-grained annotations for each frame. The data from the forward direction were used for the different augmentations, and the data from the backward direction for testing purposes.

### 3.3. Augmentation

Regarding image data augmentation, the categories and strengths of augmentations are specified. Comparable conventional and semantic augmentations were determined as shown in Table 1.

All conventional augmentations were performed using imgaug [3].

Regarding strengths, the five aforementioned categories are defined as: weak, weak-medium, medium, medium-strong, strong. In a first step, the conventional augmentations were applied to a very small dataset, which consists of ten hand-selected pictures with diverse content. The augmentations were applied separately in five different strengths. As most publications do not openly discuss parameters used for augmentation, these parameters were defined at a rough estimate while considering “best practices”. After this initial step, the LPIPS score between the ten frames and the ten augmented frames was calculated.

The semantic augmentations were prepared in a blender and parameterized so that the results match the mean LPIPS score of the conventional augmentation. This evaluation was based on the same small ten-frame dataset taken from the training data.

Due to the nature of creating semantically augmented images, much time went into the creation and rendering of the augmentations for each parameter. For many parameters, multiple edits are necessary in the domain to achieve the desired look and match the LPIPS score. The creation of semantically augmented images, therefore, is always much more complex than with conventional forms of image augmentation. Furthermore, the complexity of the changes in the semantic domain is always influenced by the parameter and the domain itself. In contrast, for conventional augmentations, it is less complex to manipulate domains and parameters as filters are used.

With the parameters for the five strength categories for both conventional and semantic augmentation determined in that way, the conventionally augmented dataset was calculated using imgaug, and the semantically augmented dataset was rendered using a blender. In total, 10,291 frames plus their fine grading annotations were rendered in blender. These consist of 251 unaugmented forward frames and 10,040 augmented frames, as seen in the equation below. Additionally, another 10,040 frames were provided through conventionally augmenting the forward frames per category and strength. This compares to other synthetic datasets such as “Virtual Kitty” [14].

$$\begin{array}{c} \underset{\begin{array}{c} unaugmented\\ frames \end{array}}{\underset{︸}{251}}+\underset{augmentedframes}{\underset{︸}{\underset{\begin{array}{c} unaugmented\\ frames \end{array}}{\underset{︸}{251}}*\underset{categories}{\underset{︸}{8}}*\underset{strengths}{\underset{︸}{5}}}}=\underset{totalframes}{\underset{︸}{10,291}} \end{array}$$

### 3.4. Network and Training

For the evaluation of the semantic augmentations, a DCNN with eleven different datasets was trained, composed of five datasets per augmentation type (each representing one augmentation strength), plus one combined dataset with semantic and conventional augmentations. First, training was performed on the conventionally augmented datasets, then on the semantically augmented datasets and lastly on the combined dataset. The eleven trained models were then tested on both, real and synthetic data.

Since the hardware resources of this project were limited, but it is necessary to work with high-resolution images at the same time, an efficient high-performance network was required. High-resolution image data are important because having as much information as possible in the images allows a meaningful comparison between semantic and conventional augmentations. After testing different networks, it was decided to use AdapNet developed by Valada et al. [2]. This network was originally designed to perform semantic segmentation on data from several different modalities, but since it was developed specifically for autonomous driving and performs excellent on less powerful hardware, it appears well suited for the tests. AdapNet’s capability to use different modalities simultaneously as inputs for the network is not used for this work.

AdapNet is based on ResNet-50 [21], but minor changes have been made, which lead us to use AdapNet instead of ResNet. First of all, another additional convolutional layer with a 3 × 3 kernel size is used in AdapNet at the start, allowing the network to learn more high resolution features. This is advantageous because the more fine details of the image data can be taken into account, the better the semantic augmentations can be distinguished from conventional ones. In addition, the output of AdapNet is of higher resolution than the output of ResNet, which is significant as the automatically rendered annotations using a blender are very fine-grained.

The rendered images, which are used during training and testing, have a resolution of 1600 × 1200 pixels. Training is performed on four NVIDIA GeForce GTX 1080Ti graphic cards. The training datasets are composed of the 251 original rendered images plus eight different augmentation types performed on the original synthetic data, which sums up to 2008 additional augmented images per dataset, resulting in the total dataset size of 2259 images per augmentation type and strength. While training, a validation dataset consisting of 100 real images is used, and validation is performed every 10 steps. As mentioned before, real image data are used in order to mitigate possible side-effects imposed by generating our own benchmarks, where the data may be biased towards semantic or conventional augmentation. Total training time for all models is 200 steps.

## 4. Results

After finishing training, eleven different models of AdapNet are obtained. These eleven models consist of two models (one trained with conventional augmentations and one with the semantic augmentations) per augmentation strength category (weak, weak-medium, medium, medium-strong, strong) plus one model with strong augmentations trained on a combined dataset of conventional and semantic augmentations.

**Figure 4:**
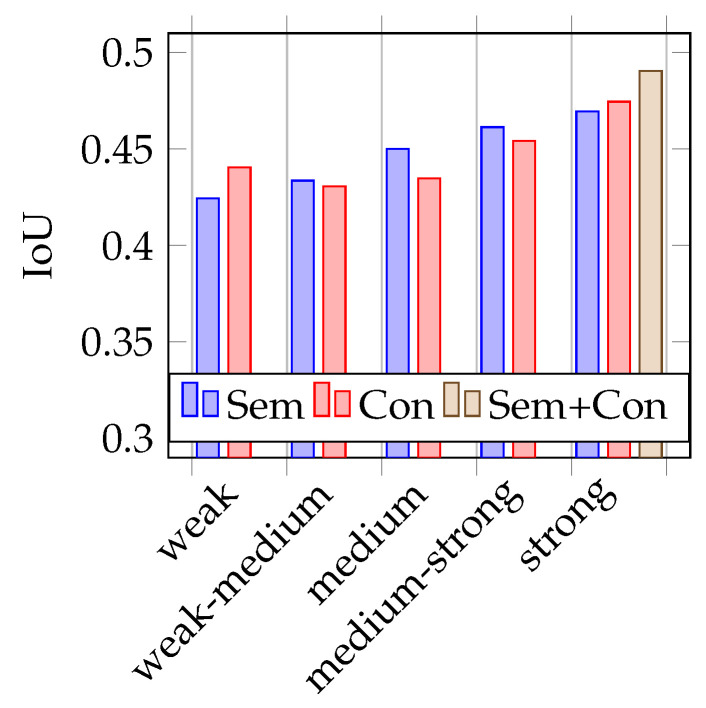
Mean IoU on real data.

**Figure 5:**
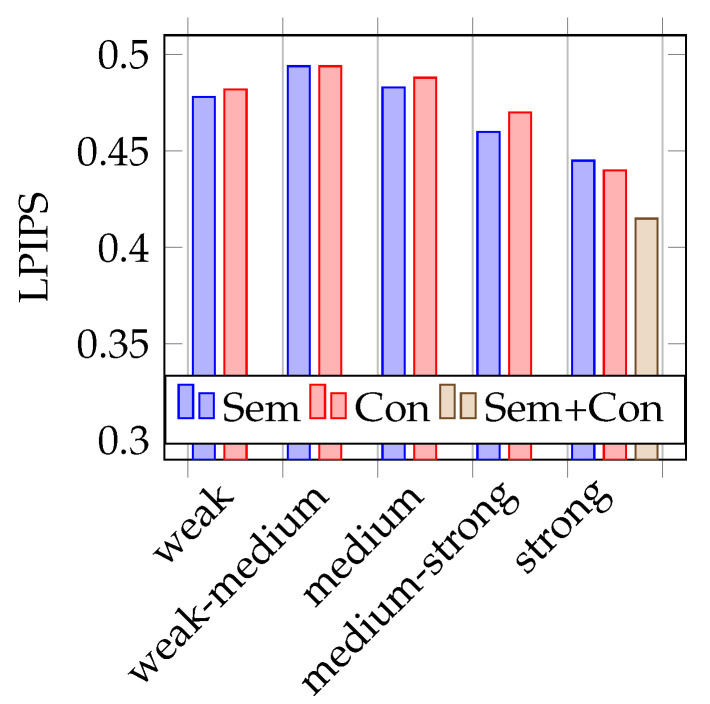
Mean LPIPS on real data.

**Figure 6:**
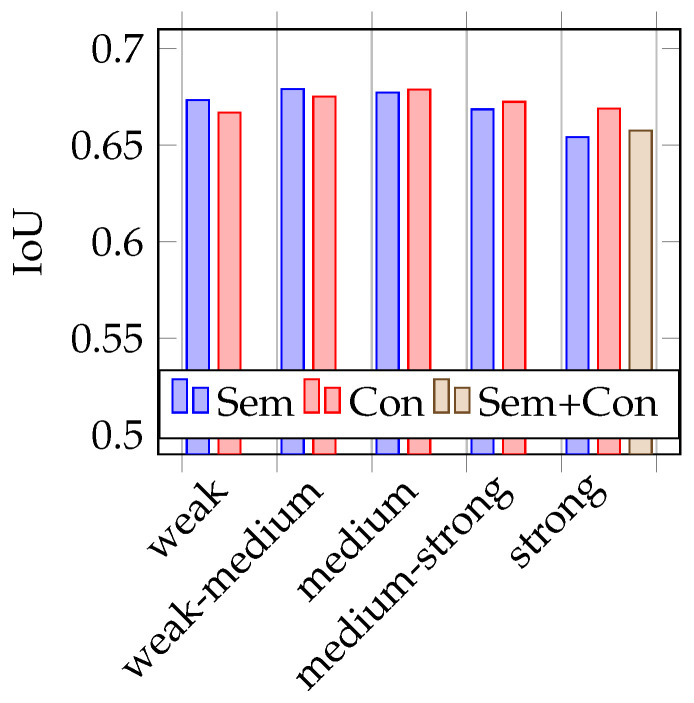
Mean IoU on synthetic data.

**Figure 7:**
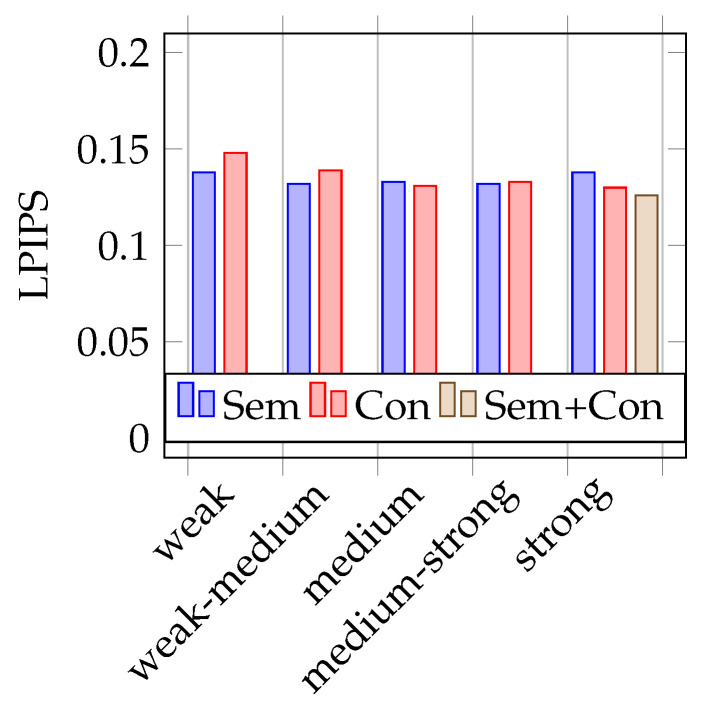
Mean LPIPS on synthetic data.

As mentioned before, to test the trained models, two different test datasets are used. One containing 387 real images (similar to the validation dataset) and the other containing the 251 synthetic images, rendered from the opposite driving direction on the 3D world in a blender. During training, it already becomes apparent that the models that are trained with strongly augmented image data perform best. This is also confirmed during the test phase, as shown in the Figure 4, Figure 5, Figure 6 and Figure 7. Therefore, the above-mentioned additional model, trained on both augmentation types, is only trained on strongly augmented data.

Overall, the models trained with semantically augmented data achieve results similar to the models trained with conventional augmentation. There is no significant difference in the performance according to the data of this work. There are, however, some conspicuous features, which will be examined in more detail below.

To evaluate the difference between the models, the Intersection over Union Score (IoU) and the LPIPS metric described in the methodology chapter are used. The IoU-score has been used in image processing, and especially the fields of object detection and image segmentation, for a longer period of time [22]. Simplified, the IoU measures how similar two annotations (or image masks) are by comparing how annotated categories overlap when superimposed. This is a really simple yet efficient metric to measure the similarity of two annotated images. The higher the IoU is, the more similar two compared images are.

Table 2 shows the obtained test results of the metrics IoU and LPIPS per trained model on real and synthetic test data. These will be considered in more detail in the following sections. Furthermore, in the Supplementary Material in Tables S2 and S3, the accuracies achieved in the tests are presented per class.

### 4.1. Testing on Real Data

As shown in Figure 4 and Figure 5, the mean IoUs of the models vary between 0.42 and 0.49, the LPIPS also between 0.49 and 0.42. In terms of the recognition performance, these are rather poor results. An explanation for this will be considered in more detail later on. However, the values are suitable for the comparison of semantic and conventional augmentation. It is noticeable that the best performing strength is strong for both metrics. Conventional augmentation achieves a slightly better IoU and LPIPS score. If the lower performing strengths are considered, semantic augmentation tends to perform slightly better here. For both metrics, the model trained with a combined dataset of strongly augmented image data achieves the best results.

### 4.2. Testing on Synthetic Data

Figure 6 and Figure 7 represent the test results on synthetic test data. The mean IoUs of the models vary between 0.65 and 0.68 and the LPIPS between 0.15 and 0.13. In the tests with synthetic training data, it is more difficult to identify trends on the impact of the use of semantic augmentations than in the tests with real data. Looking at Figure 6 showing the mean IoU, it is first noticeable that it is not the model trained with strong conventional augmentation that performs best, but the model trained on weak-medium strength semantic augmented data. Overall, there is no discernible trend here with respect to augmentation strength, but it is interesting to note that the model trained with combined augmentations performs very poorly in comparison. Looking at the results using the LPIPS metric, as seen in Figure 7, no clear trend is recognizable. The model trained with combined augmentations perform best, followed by the model trained with classical strong augmented data. However, the deviations here are really minimal.

In addition to testing the trained models on unaugmented test data, further tests were performed with the models that produced the best results. For these tests, the synthetic test images are augmented with individual augmentation categories to examine specific cases that may occur in reality. The categories used are rain, fog and snow. The test images are augmented once conventionally and once semantically. Afterward, a test dataset is created per category from both augmentation types. This is to avoid that the augmentations used in the training have a one-sided influence on the tests. The test results for these augmentation conditions are shown in Table 3. It can be seen that, at least for strong augmentation, the model trained with conventionally augmented data performs slightly better. However, these differences are only marginal to non-existent for the model trained with medium strongly augmented data, which performed better on synthetic training data in the other tests. Summarized, as with the other tests, no clear conclusions based on these different augmentation conditions can be drawn for the comparison of conventional and semantic augmentation.

### 4.3. Testing the Model Trained on a Combined Dataset

As one could expect, increasing the total amount of training data by using both semantic and conventional augmentation improves the performance of the trained model. This is also shown in Figure 4, Figure 5 and Figure 7 for both data types, real and synthetic images, as well as both metrics, except IoU on the model trained with synthetic data. It is noticeable that there is only a sparse improvement compared to the best performing models trained with conventional augmentations. Considering the mean IoU on synthetic test data, the usage of both augmentation types impairs the performance of the trained model, which is shown in Figure 6. As with the previous evaluations, these deviations are so small that no valid conclusion can be drawn about the usefulness of semantic augmentation for synthetic image data.

### 4.4. Difficulties

**Figure 8:**
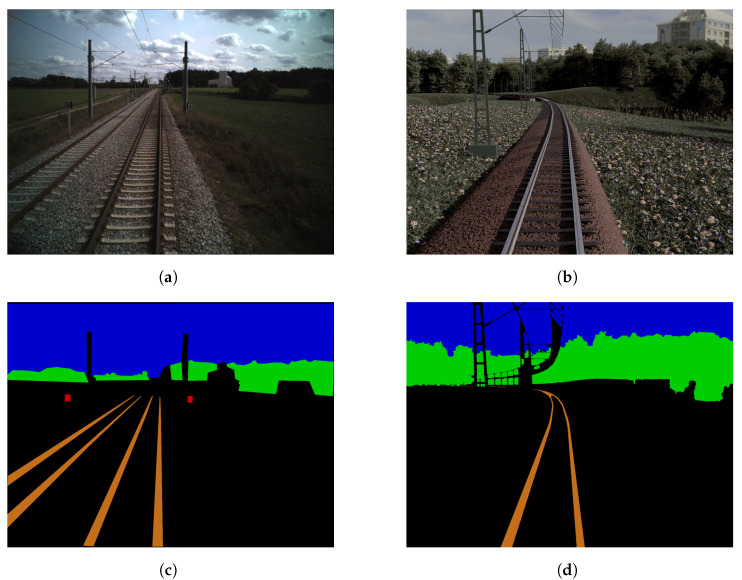
Real image (**a**) and annotation (**c**) on the left, synthetic image (**b**) and automatically generated annotation (**d**) on the right.

In the real dataset used in this paper, the images are rather roughly annotated. Compared to the synthetic images with automatically generated annotations, where details are artificially removed to obtain coarser annotations (as described in Section 3), these annotations of the real images are less accurate. A comparison between an annotation of an image from the real dataset and an annotation of a similar synthetic image is shown in Figure 8. It is clearly visible how in the real image annotation a lot of details are left out, such as the course of the rails towards the horizon or the cables. In addition, the edges that form the trees to the sky are much coarser.

**Figure 9:**
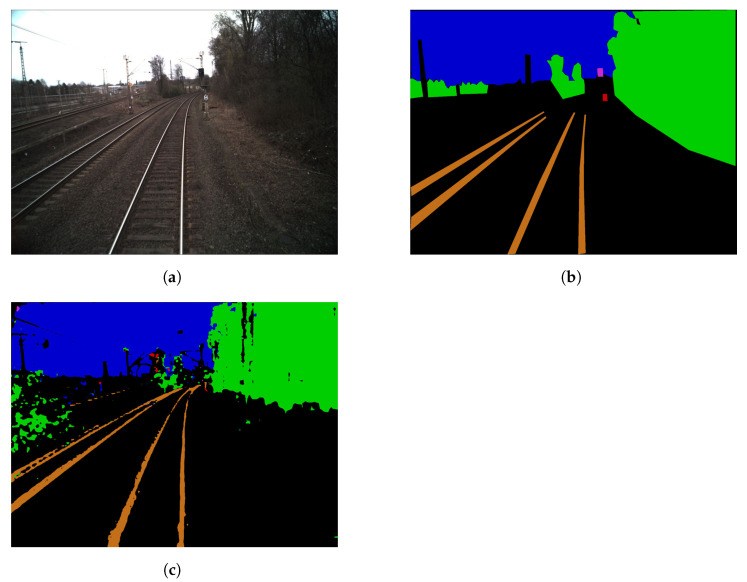
Real image (**a**) and ground truth annotation (**b**) at the top, output prediction of a trained model (**c**) below.

In testing with real data, this leads to the fact that the models trained on synthetic data annotate the real images much more detailed than the corresponding ground truth annotation of the real image definitions. This is shown in Figure 9 and of course also worsens the mean IoU. While the real image ground truth is very coarse, the trained model tries to annotate the rails a lot further towards the horizon as well as annotating cables and posts. This may be one of the reasons why the trained models perform rather poorly when testing with real images. A possible solution may be to finely annotate a small set of real data by hand, or in reverse, to reduce the details in automatically rendered annotations even more.

For testing on synthetic data, another mentionable difficulty is shown in Figure 6 and Figure 7: Since the training and test data are entirely synthetically generated, it is shown as expected that the models perform better when tested on synthetic data. This is most likely also the reason why the deviations between the different augmentation strengths and augmentation types are smaller here and thus it is harder to find clear trends.

## 5. Conclusions and Further Work

The results presented in the previous section will now be discussed in detail. As stated, the trained network models do not show significant performance improvements when trained with semantically augmented training data compared to conventional augmentations. There are slight irregularities in the performance, but no clear trend can be identified. This is true for both metrics used. In general, but especially due to the relatively high effort associated with the creation of semantic training data, it can thus be stated that a creation of semantic augmented data from a 3D environment for image augmentation is not worthwhile.

An unsurprising and rather trivial observation is that, at least with respect to the IoU, the models trained on stronger augmented data perform better when tested on more abstracted test data. This can be seen in the results generated using real test data. On test data, which is much more similar to the training data, the models trained with less augmented data, perform better. This is true for both conventional and for semantic augmentation.

To adapt the semantic segmentations to the conventional augmentations in terms of strength, the LPIPS metric is used. Since this already determines the similarity of two images based on the learned features from a convolutional layer, it is suspected that this is the reason why the results for conventional and semantic augmentation are so close. Both types of augmentation are fundamentally similar when viewed from a CNNs perspective. However, further studies on the effects of different augmentation strengths of conventionally and semantically augmented image data (according to LPIPS) would be needed to confirm this. Such an investigation could be linked to the results of this work.

Furthermore, it could be considered more closely whether semantic augmentations bring an advantage with even stronger augmentations than those that were declared as strong in this work. Furthermore, studying the same issue with different network types could provide other results that further contribute to the understanding of both semantic augmentations and the impact of using the LPIPS metric. In this context, other areas of application should also be investigated apart from the environmental recognition in the context of train runs.

Another interesting investigation for future work could be to test the effect of different augmentation methods on the robustness of semantic segmentation. For example, a suitable research question would be: Do semantic augmentations improve the robustness of semantic segmentation compared to conventional augmentation? For this purpose, another dataset could be created for benchmarking, similar to the FishyScapes [23] and ISSAFE [24] datasets. These datasets add various unexpected objects to the scenes. For example, FishyScapes adds animals to the scenes, and ISSAFE uses possible scenes before or after an accident (for example, a pedestrian running in front of the car). These two datasets cannot be used for the train ride domain, as both datasets are only concerned with the context of an urban car ride. WildDash [25] and ACDC [26] use different weather situations such as rain, fog or night to test the robustness of semantic segmentation. On the website of WildDash [27], it is mentioned that there is also the dataset RailSem19 available, which was created in the domain of a train run. However, it is also described that there is currently no benchmarking dataset of it, so it would be interesting to perform the mentioned investigation in future work and thus create a benchmarking dataset for the context of a train run.

In summary, this work would only serve as a basic lead-in to further investigate semantic augmentations for synthetic image data and is hereby welcome to initiate further work in this area.

## Figures and Tables

**Table 1:** Matched augmentation categories for the two augmentation types.

Conventional	Semantic
Rotation	Rotation of virtual camera
Snowy landscape	Snow coating
Snowflakes	Snowflakes
Rain	Raindrops + rain coating
Fog	Fog
Gaussian Blur	Defocus Blur
Change in color (hue, sat)	Change in color (manual, per object)
Exposure	Exposure

**Table 2:** Testing results.

Augmentation		Real		Synthetic	
**Strength**	**Type**	**IoU**	**LPIPS**	**IoU**	**LPIPS**
**strong**	conv. + sem.	0.491	0.415	0.658	0.126
	conventional	0.475	0.440	0.669	0.130
	semantic	0.469	0.445	0.654	0.138
**medium-** **strong**	conventional	0.454	0.470	0.672	0.133
	semantic	0.461	0.460	0.669	0.132
**medium**	conventional	0.435	0.488	0.679	0.131
	semantic	0.450	0.483	0.677	0.133
**weak-** **medium**	conventional	0.431	0.494	0.675	0.139
	semantic	0.434	0.494	0.679	0.132
**weak**	conventional	0.440	0.482	0.667	0.148
	semantic	0.424	0.478	0.673	0.138

**Table 3:** Testing on different augmentation categories (conditions).

Augmentation		IoU			LPIPS		
**Strength**	**Type**	**Rain**	**Fog**	**Snow**	**Rain**	**Fog**	**Snow**
**strong**	conv. + sem.	0.495	0.641	0.712	0.243	0.313	0.276
	conventional	0.502	0.620	0.708	0.247	0.372	0.286
	semantic	0.491	0.562	0.682	0.252	0.426	0.329
**medium**	conventional	0.504	0.483	0.689	0.252	0.467	0.320
	semantic	0.500	0.510	0.688	0.251	0.457	0.327

## Data Availability

The synthetic data presented in this study are openly available in FigShare at 10.6084/m9.figshare.14754565.v1 [28].

## Supplementary Materials

The following are available at https://www.mdpi.com/article/10.3390/jimaging7080146/s1, Table S1: imgaug parameters for conventional augmentation, Table S2: per class accuracies for testing on real data, Table S3: per class accuracies for testing on synthetic data.
