# Supplementary material for: Investigating Semantic Augmentation in Virtual Environments for Image Segmentation Using Convolutional Neural Networks
Source: J Imaging. 2021 Aug 14;7(8):146. doi: 10.3390/jimaging7080146 (PMC8404924; doi:10.3390/jimaging7080146)
Supplement: Supplementary file 1 [file jimaging-07-00146-s001.zip › jimaging-1307673-supplementary.pdf]

Supplemental material

Table S1: imgaug parameters for conventional augmentation

Parameter	weak	weak-medium	medium	medium-strong	strong
**Rotation (`Rotate()`)**[1]					
`rotate=`	±5	±10	±20	±30	±45
**Snowy landscape (`FastSnowyLandscape()`)**					
`lightness_threshold=`	220.0	200.0	180.0	160.0	140.0
`lightness_multiplier=`	1.6	2.2	2.8	3.4	4.0
**Snowflakes (`imgcorruptlike.Snow()`)**					
`severity=`	1	2	3	4	5
**Rain (`Rain()`)**					
`nb_iterations=`	1	1	2	2	3
`drop_size=`	0.05	0.15	0.15	0.175	0.20
`speed=`	0.25	0.20	0.20	0.20	0.20
**Fog (`CloudLayer()`)**[2]					
`density_multiplier=`	0.30	0.40	0.50	0.65	0.90
**Gaussian Blur (`GaussianBlur()`)**					
`sigma=`	0.7	1.0	2.0	3.0	4.0
**Hue and saturation change (`AddToHueAndSaturation()`)**					
`value_hue=`	±5	±16	±26	±68	±125
`value_saturation=`	0	0	0	0	32
**Exposure (`Add()`, `Multiply()`)**[3]					
(low) `value=`	-10.00	-20.00	-30.00	-40.00	-50.00
(low) `mul=`	0.90	0.80	0.70	0.60	0.50
(high) `value=`	10.00	20.00	30.00	40.00	50.00
(high) `mul=`	1.11	1.25	1.43	1.67	2.00

1 Additional parameters for rotation, used across all strengths: `mode=['symmetric', 'reflect']`

2 Additional parameters for fog, used across all strengths: `intensity_mean=240`, `intensity_freq_exponent=-1.75`, `intensity_coarse_scale=2`, `alpha_min=0.8`, `alpha_multiplier=0.3`, `alpha_size_px_max = 5`, `alpha_freq_exp=-3`, `sparsity=0.9`

3 Exposure was either underexposed (low values) or overexposed (high values) implemented with `OneOf()`. In both cases, `Add()` and `Multiply()` were executed sequentially (in this order) with `Sequential()`

Table S2: per class accuracies for testing on real data

augmentation strength	augmentation type	unknown	sky	vegetation	rails	lightsignal	shapesignal
**strong**	conv. + sem.	0.901859	0.866715	0.674393	0.479915	0.60813	0.323817
	conventional	0.903566	0.881005	0.651493	0.409718	0.549407	0.382491
	semantic	0.902589	0.855426	0.635115	0.44615	0.600423	0.291315
**medium-strong**	conventional	0.906037	0.821085	0.630904	0.363306	0.56132	0.383765
	semantic	0.896076	0.849188	0.628569	0.405077	0.615413	0.312844
**medium**	conventional	0.895903	0.711767	0.654485	0.328711	0.58099	0.378816
	semantic	0.895505	0.810142	0.643271	0.376876	0.591004	0.325623
**weak-medium**	conventional	0.87697	0.722992	0.681023	0.32664	0.559138	0.370551
	semantic	0.893888	0.736366	0.639194	0.383381	0.552729	0.346852
**weak**	conventional	0.887158	0.821949	0.650313	0.310993	0.526335	0.373803
	semantic	0.895476	0.742872	0.65687	0.341115	0.522407	0.309018

Table S3: per class accuracies for testing on synthetic data

augmentation strength	augmentation type	unknown	sky	vegetation	rails	lightsignal	shapesignal
**strong**	conv. + sem.	0.959204	0.975528	0.94564	0.863383	0.205275	0.24444
	conventional	0.96062	0.98308	0.950137	0.843578	0.298865	0.276999
	semantic	0.956622	0.97619	0.931133	0.877281	0.175111	0.249588
**medium-strong**	conventional	0.963019	0.980189	0.951953	0.843028	0.305432	0.278935
	semantic	0.958989	0.981044	0.938015	0.866319	0.245828	0.281934
**medium**	conventional	0.962327	0.980234	0.957216	0.834508	0.351321	0.306494
	semantic	0.961221	0.981339	0.940074	0.842526	0.304305	0.31435
**weak-medium**	conventional	0.961438	0.979488	0.958355	0.820607	0.349085	0.314363
	semantic	0.961527	0.97854	0.951733	0.835526	0.344684	0.301356
**weak**	conventional	0.962511	0.960248	0.945188	0.819543	0.375075	0.300725
	semantic	0.958129	0.978256	0.958477	0.827652	0.304782	0.291868
